# A case study on barriers to the research implementation of a novel technology in an academic medical center

**DOI:** 10.1371/journal.pdig.0001014

**Published:** 2025-10-03

**Authors:** Mechelle Sanders, Kevin Fiscella, Jack Chang, Alain LeBlanc, Peter Veazie

**Affiliations:** 1 Department of Family Medicine, University of Rochester, Rochester, New York, United States of America; 2 Clinical and Translational Science Institute, University of Rochester, Rochester, New York, United States of America; 3 Department of Public Health Sciences, University of Rochester, Rochester, New York, United States of America; Yonsei University College of Medicine, KOREA, REPUBLIC OF

## Abstract

Natural Language Processing allows extracting unstructured text data from electronic health records (EHR), but historically required extensive coding and expertise. Amazon Comprehend Medical (ACM) offers a scalable solution for mining EHR data without extensive natural language processing expertise. This case study examined barriers and facilitators to implementing ACM in an academic medical center. We reviewed correspondence regarding ACM implementation between study investigators and respective experts within the medical center. We qualitatively coded the correspondence for barriers and facilitators using the Consolidated Framework for Implementation Research (CFIR) framework as a guide. Key findings included the involvement of non-traditional stakeholders in the approval process and unexpected limitations of anticipated facilitators. The study revealed that implementing novel technologies like ACM in academic medical settings requires careful consideration of safety protocols, which may slow adoption. Our findings can guide research teams in navigating the implementation of similar technologies, balancing innovation with necessary safeguards.

## Author summary

This study describes the challenges and supports involved in using a new computer tool that can read and process written language for research in an academic medical center. Understanding these factors before introducing the tool is especially important when researchers are working on major grants, where they must make careful choices about how to use limited resources while still showing progress.We reviewed emails and messages between the research team and medical center experts about how to put the tool in place. We then organized this information to identify what helped and what made the process more difficult, using an established framework to guide our analysis.We found that people not usually involved in research decision-making were unexpectedly part of the approval process, and some supports we thought would help did not work as planned. We also found that bringing new technologies into research requires additional attention to safety measures beyond the usual protections, which can slow adoption.These findings provide practical guidance for research teams interested in using new technologies. They highlight the need to balance innovation with safeguards that protect data quality and participant safety.

## Introduction

Natural Language Processing (NLP) provides a means for extracting unstructured textual data from electronic health records (EHRs) [1]. Historically, NLP has required extensive development of coding algorithms for basic keyword/ dictionary-based extraction to more advanced concept extraction. Developing suitable codes and applying them is labor intensive and requires expertise in NLP [2].

In 2018, Amazon introduced Amazon Comprehend Medical (ACM). ACM uses deep learning to categorize information from unstructured EHR data, including clinician notes into relevant structured entities. At the cost of a penny or less for every 100 characters of analyzed text and detailed data extraction within minutes, ACM potentially represents a scalable and cost-effective means for mining previously unusable EHR information [3]. Plug and play technologies such as ACM present a compelling case for an easy-to-use platform to extract information for research teams without robust NLP support. Several studies are beginning to emerge that speak to the uptake, validation and confirmation of the data output of ACM [4–6]. However, the actual implementation of the platform has not received attention. For example, privacy in cloud-based environments is an important concern with which academic health centers and universities contend [7]. The most significant concern with using any cloud-based service, especially for medical data, involves ensuring the privacy and security of patient information and compliance with regulations governing the handling, processing, and sharing of medical data [8]. Data breaches or unauthorized access could lead to exposure of sensitive health information, violating regulations like HIPAA in the U.S [9]. Ensuring that data are encrypted, both during transfer and in storage, is critical. The lack of protocols to attend to these issues may slow or inhibit the ability of research teams to use such technology despite its promise. This can be especially important in the context of high-stake research grants where investigators will need to make decisions amid resource constraints and demonstrate productivity [10].

Our aim for this case study was to identify the barriers to implementing the ACM in an academic medical center. We used the Consolidated Framework for Implementation Research (CFIR) [11] to understand implementation successes and failures. Next, we discuss how our implementation expectations differed from what we observed. Finally, we discuss the implications and offer suggestions to address the unique implementation factors related to novel technologies in academic medical settings.

## Methods

### Setting & participants

We conducted the study at a university with a very high research activity (R1). We reviewed meeting minutes and email correspondence regarding ACM implementation between study investigators, the bioinformatics department, the institutional review board, the information security office, and the privacy office within the medical center. The correspondences occurred between August 2019- February 2020.

Our research coordinator (RC) facilitated recruitment. Six people were eligible and recruited to participate in the study. This included all the people the study team contacted about implementing the project.

### CFIR coding

We combined anonymized email correspondences and sorted them chronologically. We then divided the correspondence data into five segments based on who the correspondence was with (e.g., bioinformatics department). The mean number of words in each segment was 1387 (range 452–2009). We retrospectively analyzed the factors influencing implementation using a group deliberation approach, mapped against the CFIR. The CFIR includes 5 domains and 67 constructs that have been shown to influence implementation success, namely, intervention characteristics (e.g.,; adaptability, design quality, and cost), the outer setting (e.g., external policy, peer pressure), the inner setting (e.g., culture, climate, and readiness for implementation), the characteristics of individuals (e.g., knowledge and beliefs about the intervention) and the process of implementation (e.g., planning, engaging, executing and reflecting). In total, we selected 13 constructs a priori across four domains [11]. We excluded items that were not applicable we did not think applied to the study. For example, we did not require physical space to implement the technology and excluded that code. We found the original CFIR domains and associated constructs to be adequate for codes and we did not add any additional codes.

### Analyses

We used the CFIR framework as a guide to understanding the barriers and facilitators to implementation. To compare the relative contribution of each CFIR domain, we coded each construct as a facilitator, as a barrier, or as having no influence (neutral) on implementation. First, each team member independently coded a subset of the same correspondences or communications using the CFIR constructs. We compared coded segments, discussed discrepancies, and refined the definitions to the codes as needed.

We (KF, MS, PV) met 3 times to review and discuss the CFIR definitions and come to a consensus on the meaning of each code and how and if they were relevant to our project. Once we reached a final consensus on the codes, we developed a codebook and practiced applying the codes with a sample of a segment together (500 words).

Subsequently, each team member independently coded segments and entered the codes they observed in the data into a spreadsheet that contained each of the CFIR constructs. At least two coders were assigned to each segment. The final codes were summarized and collated for analysis. Discrepancies in coding were resolved through consensus. We did not test intercoder reliability and instead used consensus to address any coding differences [12].

Finally, using group consensus, we reviewed the CFIR constructs and retrospectively indicated the impact we *expected* each would have prior to implementation. We compared our expected outcomes to what was observed in the coded data. We wrote a high-level summary of our findings for each CFIR domain/construct (Table 1).

**Table 1:** CFIR definitions and findings.

Domain	CFIR Construct	Definition	Our Findings	Impact
**Intervention Characteristics**	***Innovation Source***	The group that developed and/or visibly sponsored use of the innovation is reputable, credible, and/or trustable.	We did not feel we had sufficient control to safely implement the technology. We could not guarantee the real-world data we planned to load would not contain any PHI. At the time, the University was finalizing data security policies PHI in cloud environments.	barrier
	***Innovation Trialability***	The innovation can be tested or piloted on a small scale and undone.	We could not test the software with real-world data until the data security issue was resolved.	barrier
	***Innovation Adaptability***	The innovation can be modified, tailored, or refined to fit local context or needs.	Our research goals required an additional service from AWS Sagemaker Studio. At the time, it was unavailable in our region. We were unaware of the limitations this may have had on product functionality.	barrier
	***Innovation Complexity***	The innovation is complicated, which may be reflected by its scope and/or the nature and number of connections and steps.	We were unaware that we needed to upload data into a MySQL database before the start of the study.	barrier
	***Innovation Design***	The innovation is well-designed and packaged, including how it is assembled, bundled, and presented.	The demonstration of Amazon Web Services (AWS) Certificate Manager (ACM) on the company’s website significantly influenced our choice of software, particularly due to its promotion as cost-effective and user-friendly.	facilitator
	I***nnovation Cost***	The innovation purchase and operating costs are affordable.		facilitator
**Outer Setting**	***Partnerships & Connections***	The Inner Setting is networked with external entities, including referral networks, academic affiliations, and professional organization networks.	The University’s BAA was with Amazon Web Services (AWS) not ACM, specifically. We did not realize this, nor did we review the BAA ahead of time to determine if the details of the agreement jived with our proposed study.	neutral
**Inner Setting**	***Information Technology Infrastructure***	University relationships with external vendors; methods procedures, protocols, etc.	The University was developing a cloud policy and a security process at the same time we were trying to implement the project. Therefore, the protocols were not yet in place.	barrier
	***Available Resources***	Resources are available to implement and deliver the innovation.	Academic IT had knowledge and were able to provide us with a 10-line demo script to pull data from an AWS S3 bucket, perform its analysis, and dump the data in text/json form.	facilitator
	***Communications***	There are high quality formal and informal information sharing practices within and across Inner Setting boundaries (e.g., structural, professional).	Our project required additional administrative approvals beyond the traditional IRB review.	neutral
	***Culture***	There are shared values, beliefs, and norms across the Inner Setting.	The University prioritized data security and avoiding risks, whereas our study was driven by innovation.	barrier
**Implementation Process**	***Teaming***	Join together, intentionally coordinating and collaborating on interdependent tasks, to implement the innovation.	We found good mission alignment. There were planning across the institution--though not always in sync with each other.	facilitator
	***Implementation Climate***	The extent to which the Inner Setting has an implementation climate.	We failed to identify critical contextual barriers.	neutral

### Ethics statement

The University of Rochester Institutional Review Board approved the study. Participants provided eConsent via REDCap.

## Results

Five participants (83%) consented to using their anonymized email correspondences in the study. To protect the anonymity of the participants due to the small sample size, we did not include any personally identifiable information nor direct quotes.

**Fig 1:**
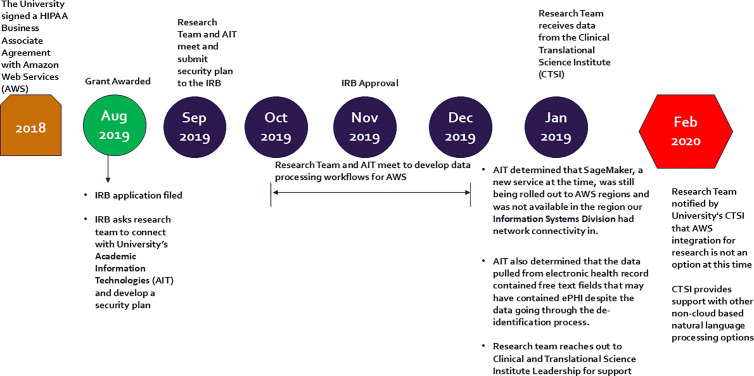
Study Timeline. This timeline illustrates key events influencing implementation from the time the grant was awarded. Major milestone including grant acquisition, IRB approval, and key decisions by institutional leadership are indicated by circles. The green circle marks the start of implementation efforts by the study team, while the red hexagon indicates the end of these efforts. Abbreviations: AWS (Amazon Web Services); IRB (Institutional Review Board); AIT (Academic Information Technologies); CTSI (Clinical and Translational Science Institute).

**Fig 2:**
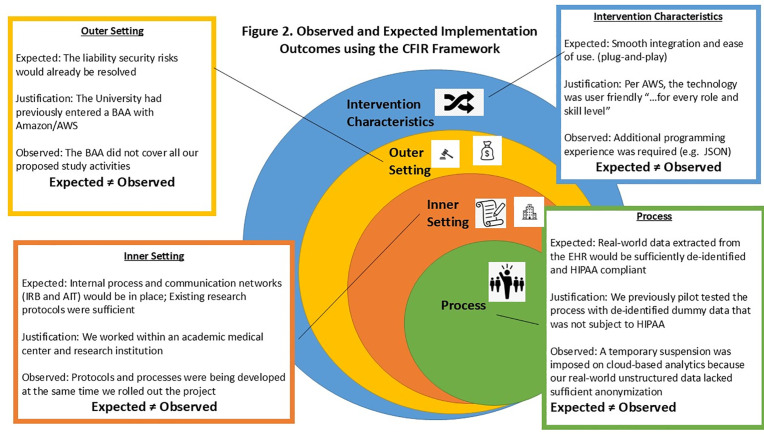
Observed and Expected Implementation Outcomes using the CFIR Framework. This figure summarizes both observed and the study team’s expected implementation outcomes, organized according to the Consolidated Framework for Implementation Research (CFIR) domains. Observed outcomes are based on data collected during the implementation period, while expected outcomes reflect initial project goals and benchmarks. Outcomes are categorized by relevant CFIR constructs, including intervention characteristics, inner setting, outer setting, characteristics of individuals, and implementation process. Differences between observed and expected outcomes are highlighted to identify key facilitators and barriers encountered during implementation. Abbreviations: CFIR (Consolidated Framework for Implementation Research).

A description of the CFIR constructs and their influence on the implementation of ACM at the medical center are presented in Table 1. Overall, 46% of the constructs were barriers (6/13), 31% were facilitators (4/13), and 23% (3/13) had no impact. ACM was only partially implemented over a 2-year timeframe, Fig 1. We found many of the intervention’s characteristics that we expected would facilitate implementation turned out to have limitations or were barriers, Fig 2.

### Expected vs. observed outcomes by CFIR domain

#### Intervention characteristics.

Given we were unable to test the trialability of ACM with real patient data, we cannot attest to its real-world trialability.

At the time, the technology was prospectively testable on the ACM website (design quality and packaging) and required minimal manual database management experience. It was marketed by ACM as a low-cost natural language processing (NLP) platform that could be used to categorize information from clinician notes and patient health records and produce predictive models using machine-learning techniques. It was marketed as scalable at a minimal cost of a penny or less for every 100 characters of analyzed. Our team was able to use the platform on the company’s website with ease. No previous programming knowledge was necessary. Our team expected this would facilitate implementation.

However, we found implementing the purchased ACM program was highly complex. It required support outside the research team from a Masters level data programmer with MySQL experience. The programmer had to write and run Python code in the ACM cloud development environment. This created a barrier to implementation, as we had anticipated the ACM environment would be plug-and-play.

#### Outer setting.

Prior to implementing our study, we learned the medical center had entered into Business Associate Agreement (BAA) with Amazon Web Services (AWS) that covered numerous products including ACM. Fig 2. We had expected this agreement would serve as a facilitator. However, we later learned that entering the BAA was a necessary but insufficient condition for conducting the study. At the time, the medical center had not uploaded any real patient data into ACM. Our study aimed to determine the feasibility of uploading patient data to ACM to develop a characterization profile of patients seen in our Emergency Departments. We observed that this was not in the BAA.

Notwithstanding the BAA between the medical center and AWS, we were unaware that the medical center had placed a moratorium on all cloud-based analytics then. Thus, we failed to identify key contextual barriers.

#### Inner setting.

Several departments in the medical center were independently developing policies for cloud-based applications. For example, some leaders worked on protocols around liability for patient-related data and others for consented subjects’ data. Unfortunately, determining processes for approval to use ACM for the study and from whom was not immediately self-evident to the research team.

#### Implementation outcomes.

We did not fully implement the ACM technology. We purchased and loaded the program but could not run it with real-world data.

## Discussion

Our study aimed to retrospectively describe the determinants of implementing a novel technology at an academic medical center for a research study. We could not fully implement the technology as planned. Our findings show that all the implementation factors we expected would be facilitators, turned out to be barriers or came with limitations.

For many factors, it was not *if* a particular implementation factor was in place, but *how* the factor was currently being used. The research ecosystem at an academic medical center is complex. When seeking approval to conduct research on innovative technologies, researchers may need to include stakeholders that are not traditionally involved. For example, the medical center’s legal experts can be essential advisors when planning to implement new technologies that interact with external sources [13].

The strengths of our study include the broad views of multiple stakeholders. Our findings are based on longitudinal correspondences, thus broadening our understanding of the degree to which each implementation factor played a role and how those factors may have evolved or changed over time. Highlighting the fact that implementation is not a linear process.

The CFIR proved to be a helpful framework for retrospectively identifying unique factors that impact success before and during the implementation of ACM. We recommend researchers use the CFIR to prospectively determine potential barriers to implementing novel technologies before roll-out [14].

## Implications

We doubt our experience is uncommon when large organizations adopt novel technology with multiple layers of approval. It often takes time to evaluate potential risks, develop mitigation policies, and communicate policies and processes for approval. These unknowns can pose a risk to research with tight timelines. Therefore, research teams working at the intersection of novel technology and implementation science should consider starting with smaller less “risky” projects before full implementation. They can then scale-up as they test and learn. This can avoid potentially costly setbacks associated with failed implementation. It will also be important to make sure there is a person on the team who has expertise and access to any legal or business agreements that may be in place *prior* to implementation. Business contracts may contain data security or privacy clauses that protect the University from risk whilst halting research use. Moreover, research teams do not want to risk inadvertently putting the University at risk of violating contract terms they may be unfamiliar with.

Our study adds to the implementation literature around novel and innovative technologies. As, Braithwaite *et al*. note, a step-by-step approach to implementation is not feasible [15]. Especially, for researchers on the brink of early adoption. The CFIR was a useful framework for identifying changes in pre- and post-implementation factors over time. At the time of implementation, our medical center did not have protocols for researchers interested in implementing cloud technologies. Since then, University Information Technology (IT), Information Systems Division (ISD), and the Office of Research IT have formed a cloud governance model for the entire University, Medical Center, and research communities. This project intake process provides consultation from a central cloud architecture team to examine proposed cloud solutions, along with a documented Shared Responsibility Model and a Public Cloud Acceptable Use Agreement. In addition, a central cloud operations team monitors cloud configuration issues, vulnerabilities in cloud resources, and data security issues. This team works with cloud account owners to resolve any issues that put the University or its data at risk. Authentication and access control to cloud services have also been centralized to use Entra ID/Active Directory credentials.

## Limitation

Despite our study’s strengths, there are some important limitations. Our findings are for one university and one technology. This may not be generalizable to all innovation implementation studies. Second, the CFIR may have missed important implementation factors that we did not include. Third, we were limited to only 5 informants and one data source. One person did not consent to have their data included in the study. Their involvement was limited to a single brief email exchange with the team, which connected us to another participant that consented and answered the question we posed to the non-consenting person. We believe this missing data had minimal impact on our results. However, our findings may have limited applicability due to small sample size and type of data collected (i.e., correspondence data only).

It is worth noting that institutional policies regarding novel research technologies were still in development at the time of our study. Therefore, we are unable to triangulate or findings or implement the ACM technology.

## Conclusion

We hope that our findings will lead to a better understanding of the nuances of implementing innovative technologies in academic medical centers. Our findings can guide decisions made pre, during, and post-implementation. Our results show consultation among multiple and non-traditional stakeholders in the research process needs to be considered when designing and implementing a study using novel technology.
